# Natural products in treating diabetic kidney disease: a visualized bibliometric analysis

**DOI:** 10.3389/fphar.2025.1522074

**Published:** 2025-05-19

**Authors:** Tianlong Liu, Xiaolin Li, Yidan Chen, Jinhu Li, Rong Wang, Yi Ding, Minna Liu

**Affiliations:** ^1^ Department of Pharmacy, The 940th Hospital Joint Logistics Support Forces of PLA, Lanzhou, China; ^2^ Department of Pharmacy, Xijing Hospital, Fourth Military Medical University, Xi’an, China; ^3^ Fundamental Medical Science Research Laboratories and Department of Nephrology, The 940th Hospital Joint Logistics Support Forces of PLA, Lanzhou, China

**Keywords:** natural products, diabetic kidney disease, bibliometric analysis, citespace, VOSviewer

## Abstract

**Background:**

Diabetic kidney disease (DKD) is one of the most significant complications in diabetic patients, yet current therapeutic options are limited. The advantages of natural products in treating chronic diseases have increasingly garnered attention. This study aims to map the landscape of natural products in DKD and provide new insights for future research in this field.

**Methods:**

Literature retrieval was conducted through the Web of Science. CiteSpace and VOSviewer were employed to conduct visual analyses of these papers.

**Results:**

A total of 523 literature were obtained, originating from 655 institutions across 40 countries/regions and involving 3,116 authors. These literature were published in 178 journals. The results indicate that China leads in this field, with Li Ping contributing the most publications. Zhang Lei’s work has been cited the most. Journal of Ethnopharmacology is the most popular journal. The paper with the highest average annual citation rate is authored by Tang, GY. Keyword analysis reveals that systematic biological approaches such as network pharmacology, molecular docking, and gut microbiota have become hotspots in this field.

**Conclusion:**

Natural products exhibit positive pharmacological activity and therapeutic value in the treatment of DKD. Extensive cooperation and communication among countries, institutions, and authors still need to be strengthened to promote basic research and clinical applications of natural products. Besides, the deep integration of network pharmacology with artificial intelligence and big data represents a hot topic and trend in future research on natural products against DKD.

## Graphical Abstract

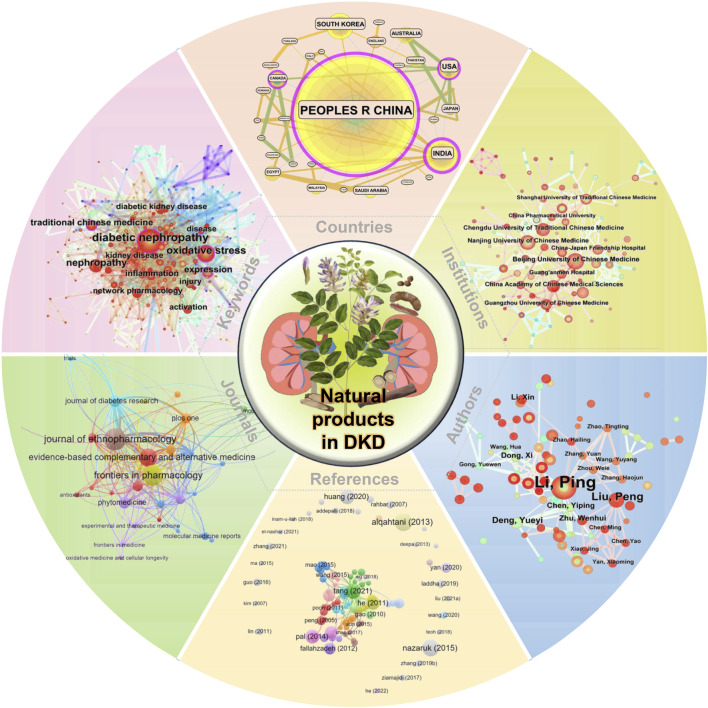

## 1 Introduction

Diabetic kidney disease (DKD) is one of the most serious microvascular complications caused by diabetes, characterized by proteinuria, edema, decreased glomerular filtration rate, and hypertension, and is emerging as a major public health problem (Herrington and Haynes, 2024). DKD typically has an insidious onset, with nonspecific early symptoms that often lead to delayed diagnosis and inadequate early intervention due to low clinical suspicion (Gnudi et al., 2016). In terms of pathogenesis, DKD mainly manifests as metabolic disorders, hemodynamic abnormalities, inflammation, and oxidative stress (Zhou et al., 2024). Pathologically, early-stage DKD is characterized by thickening of the basement membrane of glomeruli, expansion of mesangial cells, and damage to podocytes and renal tubular cells (Zhong et al., 2024). In advanced stages, it shows glomerular sclerosis and fibrosis of the renal interstitium (Zheng et al., 2021). Clinically, patients frequently present with poor digestion, nausea and vomiting, generalized itching, and fatigue (Kanasaki et al., 2024). Currently, modern medicine has no effective treatment for DKD, mainly focusing on controlling blood sugar, inhibiting the renin-angiotensin-aldosterone system (RAAS), and sodium-dependent glucose transporters 2 (SGLT2) (DeFronzo et al., 2021), improving diet and exercise, but often with poor results, severe adverse reactions, expensive treatment, not only causing great pain to patients, but also bringing heavy burdens to families and society (Georgianos et al., 2023).

In recent years, natural products have shown promising therapeutic potential in improving the clinical symptoms of DKD (Hu et al., 2023). According to the Mogensen staging system of DKD, patients in stages III-IV can benefit from effective interventions to prevent the progression of their condition, and even achieve curative effects, which is of great significance for improving prognosis (Hu et al., 2023).

Bibliometrics is the interdisciplinary science that uses mathematical and statistical methods to quantitatively analyze all knowledge carriers (Hicks et al., 2015). It is a comprehensive knowledge system integrating mathematics, statistics, and bibliography, focusing on quantitative integration (Wu et al., 2023). The application of advanced information visualization techniques and methods can provide a clear visual representation of the research development trajectory, current status, research hotspots, and development trends of a given topic (Yarmohammadi et al., 2024). In this paper, we use bibliometric methods to analyze relevant literature on natural products against DKD, and present the statistical results using CiteSpace and VOSviewer software to generate visual knowledge maps, identifying research hotspots and trends, with the aim of providing important references for future research in this field.

## 2 Methods

### 2.1 Data collection

**FIGURE 1:**
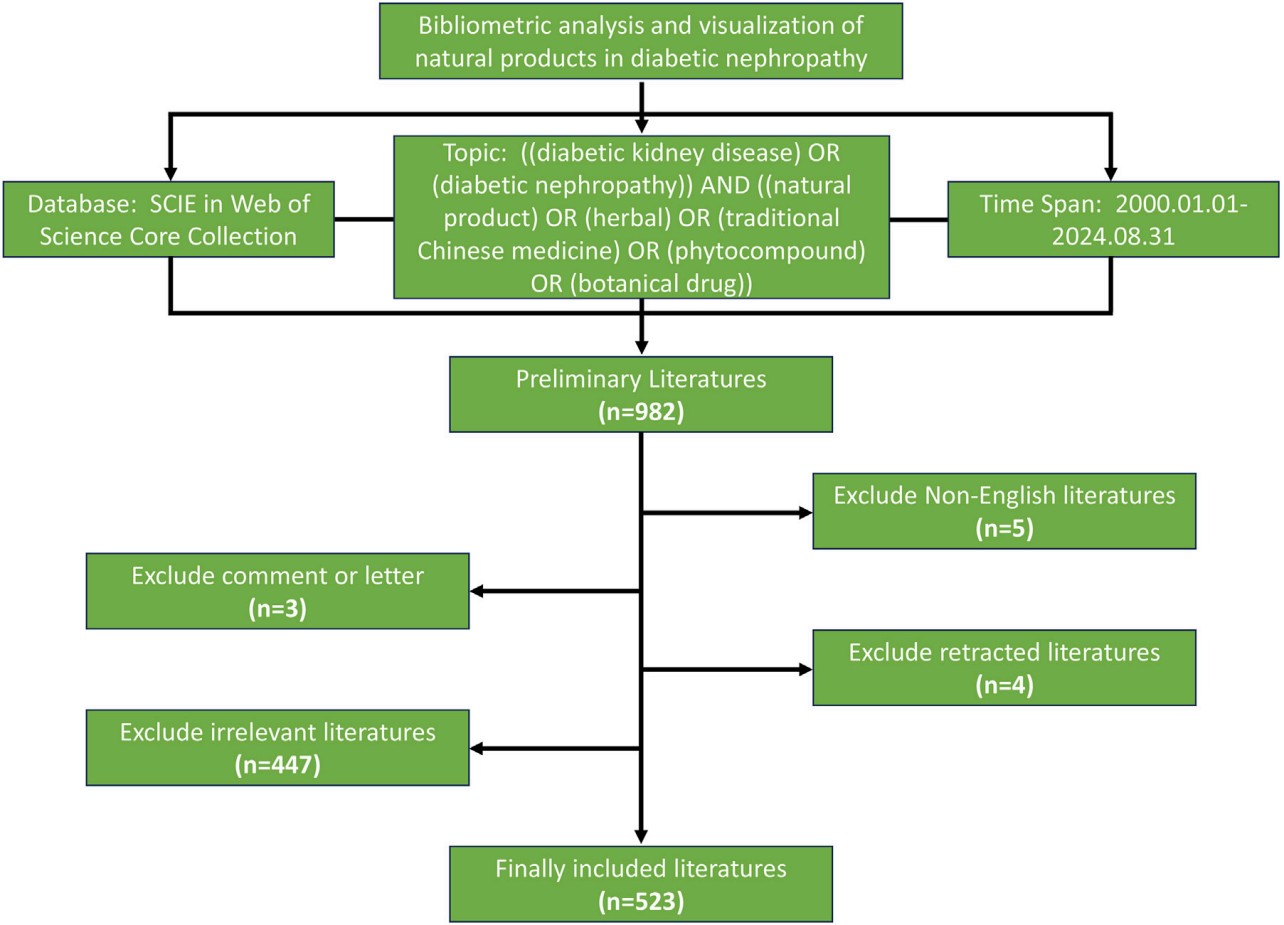
Flowchart of literature screening.

All literature were extracted from the Web of Science (WOS) Core Collection’s Science Citation Index Extended (SCIE) Database. The retrieval strategy is: “TS = ((diabetic kidney disease) OR (diabetic nephropathy)) AND ((natural product) OR (herbal) OR (traditional Chinese medicine) OR (phytocompound) OR (botanical drug))”. Since the beginning of this century, the research of natural medicine has developed rapidly, thus the search time span is from 1 January 2000 to 31 August 2024. Based on the above restriction, a total of 982 literature were obtained preliminary. The language of the literature was limited to English (Tian and Chen, 2024), and five non-English literature were excluded. The type of the literature is limited to original research and review (Liao et al., 2024), and three other types of literature were excluded, including one editorial comment, 1 letter and 1meeting abstract. Besides, four retracted literature were also excluded. Two researchers independently screened all literature based on the title and abstract. After the screening, the researchers compared the results and invited a third researcher to independently evaluate the literature for inconsistent screening results. If a literature is in dispute and a third researcher also cannot determine, all researchers will meet to discuss and decide. Before this, all the researchers were trained and clarified the general principles of literature screening to ensure the consistency of screening standards and reduce the bias. Finally, 447 irrelevant literature were excluded and finally 523 literature for this study were obtained. The retrieved literature were exported and saved as plain text files in a download. txt format. The process of literature retrieval and screening is shown in Figure 1.

### 2.2 Data analysis and visualization

Microsoft Office Excel 2021 was used to manage data and analyze the annual distribution of literature. CiteSpace (Version 6.3.3_Standard) and VOSviewer (Version 1.6.20) were used for visual analytics. The key points of analysis mainly include the country/region, institution, journal, author, keywords, and relevant co-citation information of the literature. VOSviewer is a free and open-source software used for bibliometrics and scientific visualization developed by Professor Van Eck and Waltman (Van Eck and Waltman, 2010). This tool is primarily designed to assist researchers in analyzing, visualizing, and understanding academic literature, knowledge networks, collaborative relationships, and research hotspots. CiteSpace is a software tool developed by Professor Chaomei Chen for visualizing and analyzing scientific literature (Chen, 2006). It can help researchers analyze information such as citation networks, author collaboration networks, and topic evolution, in order to identify the research trends and hotspots in the field.

## 3 Results and discussion

### 3.1 Publication output trends

**FIGURE 2:**
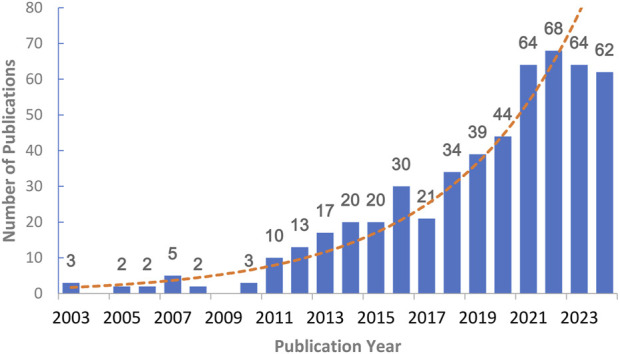
Annual publications related to natural products in treating diabetic kidney disease between 2000 and 2024. The abscissa represents the year of publication, and the ordinate represents the number of publications. Since there are no publications in 2000–2002, the horizontal coordinate of the figure begins in 2003.

The number of publications in a specific field during a certain period of time reflects the level of research enthusiasm in that field. Among the 523 papers involved in this study, there were few studies on natural products against DKD from 2003 to 2010, with only three papers per year on average. However, from 2011 to 2020, the number of published papers showed a clear upward trend, increasing from 10 papers to 44 papers per year. It is worth noting that there has been a significant increase in the number of publications in the past 4 years, basically maintaining at over 60 papers per year. This shows that the research of natural products against DKD has attracted more and more attention from the academic community in recent years (Figure 2).

### 3.2 Countries/regions and institutions distribution

**FIGURE 3:**
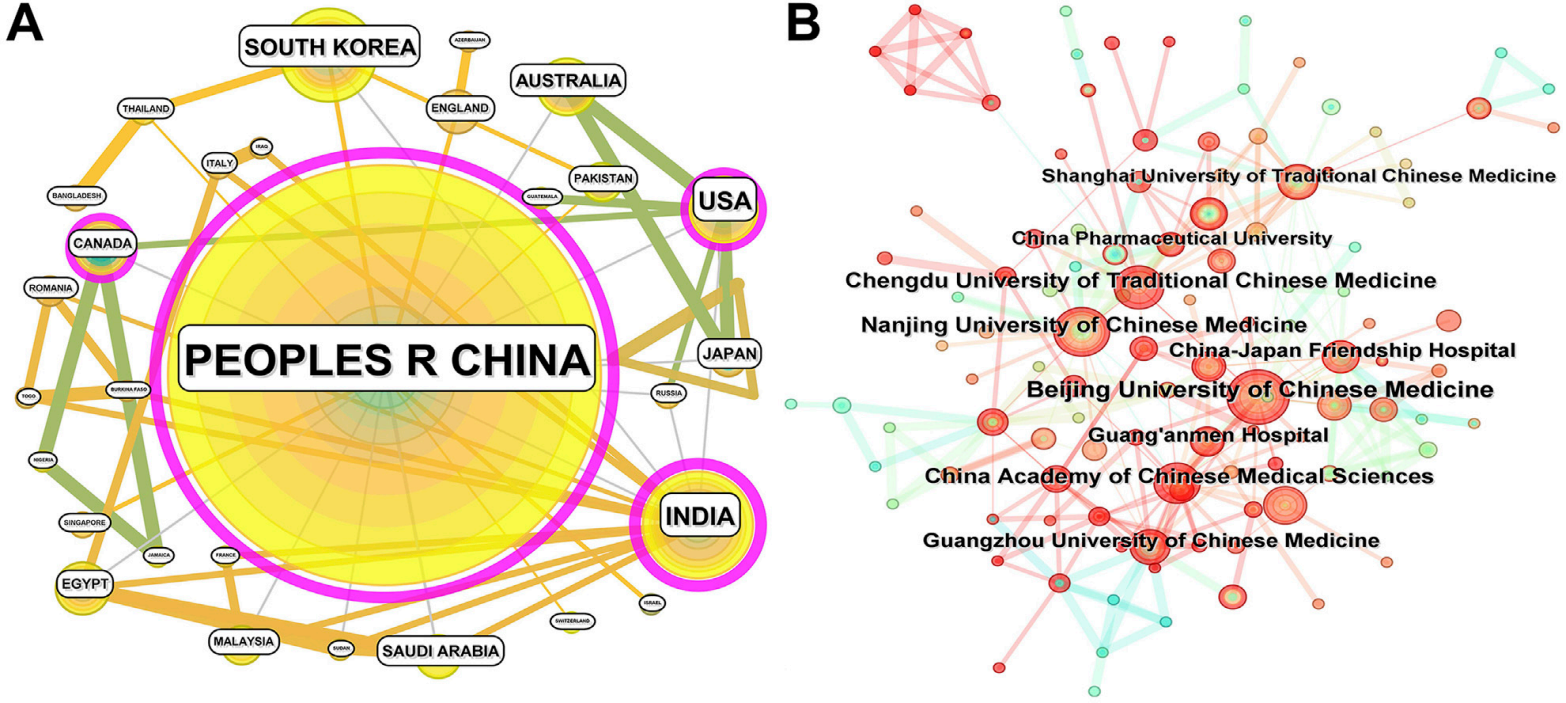
National and institutional distribution of publications related to natural products in treating diabetic kidney disease. **(A)** CiteSpace visualization map of different countries. **(B)** CiteSpace visualization map of different institutions. The diameter of the node (circle) represents the number of publications, and the larger the diameter, the larger the number of publications. The lines between nodes indicate the cooperative relationship between them, and the thicker the lines, the more frequent the cooperation.

All the 523 publications are from 40 countries/regions. As shown in Figure 3A; Table 1, China has published 437 papers, accounting for 83.56% and the centrality is as high as 0.91. It can be seen that China has an absolute advantage in the field of natural product research. The United States, India, South Korea and Australia have published 27, 24, 21 and 12 papers respectively. The network diagram shows that China has close cooperation with other countries/regions, especially with India. However, taking into account the differences in population across countries, the number of publications were standardized based on the population of each country in 2024. The results show that although China has the largest number of publications in absolute terms, the number of publications *per capita* ranks only third. Although Australia ranks fifth in the total number of publications, it has the first number of publications *per capita*, followed by South Korea. This shows that, relatively speaking, Australia and South Korea have more advantages in research of natural products in treating diabetic kidney disease.

**TABLE 1:** Distribution of publications from different countries and institutions.

No.	Country	Count	Publications per 100 million population	Centrality	Institution	Count	Centrality
1	Peoples R China	437	30.67	0.91	Beijing University of Chinese Medicine	39	0.08
2	United States	27	8.08	0.15	Nanjing University of Chinese Medicine	31	0.09
3	India	24	1.67	0.43	China Academy of Chinese Medical Sciences	29	0.06
4	South Korea	21	40.38	0.03	Chengdu University of Traditional Chinese Medicine	27	0.07
5	Australia	12	44.44	0	Guangzhou University of Chinese Medicine	23	0.05
6	Iran	8	9.33	0	China-Japan Friendship Hospital	23	0.03
7	Japan	7	5.57	0	Guang’anmen Hospital	23	0.01
8	Saudi Arabia	7	18.92	0	Shanghai University of Traditional Chinese Medicine	20	0.07
9	Canada	6	15.38	0.16	China Pharmaceutical University	19	0.02
10	Egypt	6	5.68	0.02	Changchun University of Chinese Medicine	14	0.01

Note: Publications per 100 million population = Count/Population of the country in 2024.

The results of institutional analysis indicate that the publications are mainly concentrated in China, and six of the top 10 institutions are specialized universities of Chinese medicine, which is in line with the objective law (Figure 3B; Table 1). Beijing University of Chinese medicine and Nanjing University of Chinese Medicine published the most, with 39 and 31 papers respectively. In addition, China Academy of Chinese Medical Sciences, China-Japan Friendship Hospital, Guang’anmen Hospital and China Pharmaceutical University also have outstanding performance in this field. From the results of network distribution, Beijing University of Chinese medicine has more cooperation with China Academy of Chinese Medical Sciences, China-Japan Friendship Hospital and Guang’anmen Hospital, while Nanjing University of Chinese medicine has closer cooperation with Chengdu University of Traditional Chinese medicine, Shanghai University of Traditional Chinese medicine, China Pharmaceutical University and other institutions, which may be related to the southern and northern regions. Institutions in similar regions have more convenient communication and cooperation. It should be noted that the top 10 institutions are all Chinese, but this is mainly because China has the highest total number of publications. As previously analyzed, the absolute number of publications cannot fully represent the research strength of a country or institution, and the country’s population, the number of researchers, and the number of publications in all fields should also be taken into account.

### 3.3 Authors and co-cited authors analysis

**FIGURE 4:**
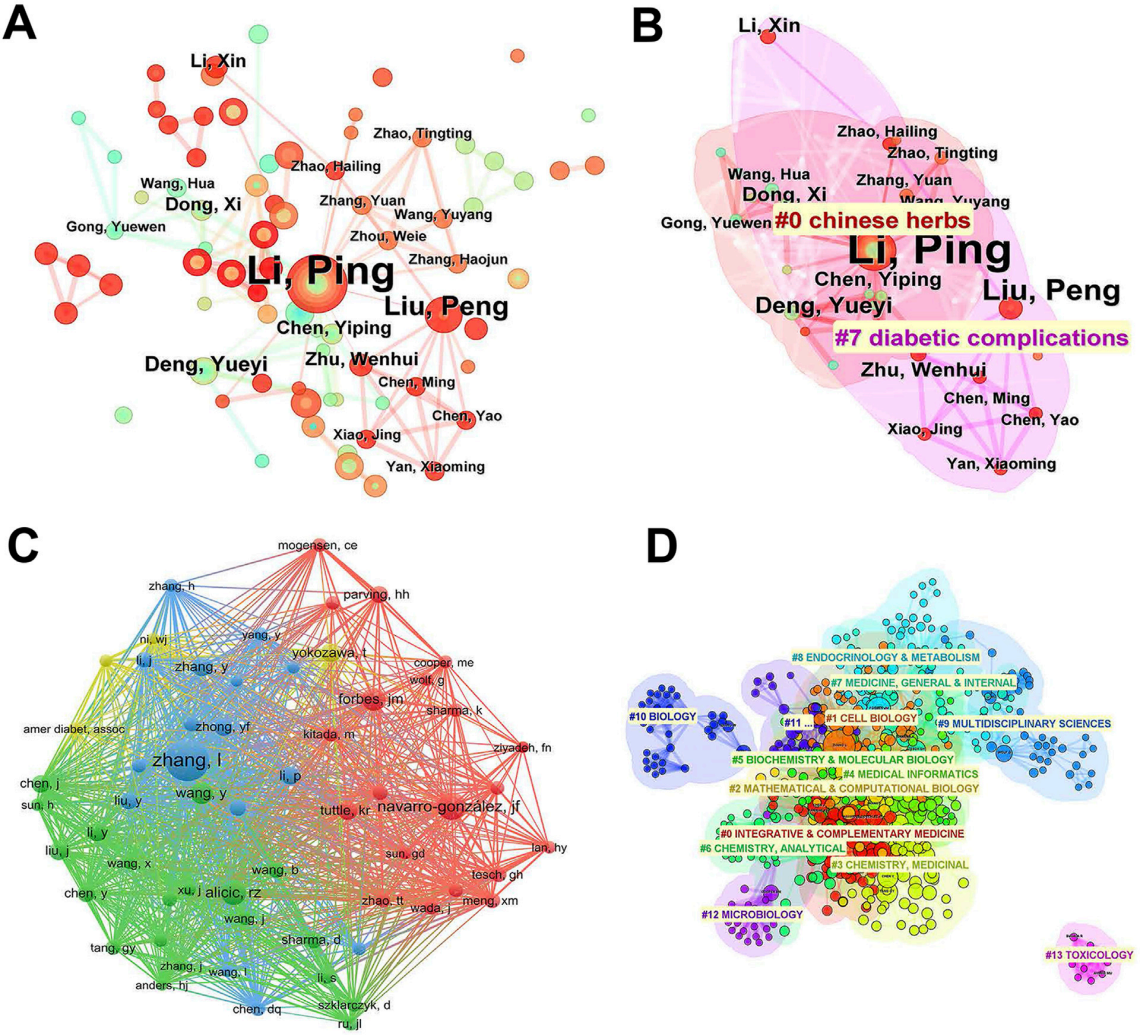
Authors and co-cited authors distribution of publications related to natural products in treating diabetic kidney disease. **(A)** CiteSpace visualization map of different authors. Node sizes are proportional to the number of papers published by these authors, and lines indicate associations between them. **(B)** Cluster analysis of the authors based on their research field. All authors were divided into two main clusters according to their research field, represented by different colors and umbrella shapes. **(C)** VOSviewer visualization map of different co-cited authors. Node sizes are proportional to the number of papers published by these co-cited authors, and lines indicate associations between them. VOSviewer classifies these co-cited authors into four main categories, denoted by red, blue, green, and yellow. **(D)** Cluster analysis of the co-cited authors based on their research field. All co-cited authors were divided into 14 main clusters by CiteSpace, represented by different colors and umbrella shapes.

A total of 3,116 authors participated in the work of these literature (Figure 4A; Table 2). The largest number of papers was published by Professor Li, Ping of the China-Japan Friendship Hospital, who published 22 papers in total, which formed a sharp contrast with other authors, indicating the leading advantage in this field. Followed by Professor Liu, Peng of Beijing Hospital of Traditional Chinese Medicine, who has published seven papers. Further cluster analysis based on the author’s research field found that these researches were mainly distributed in two fields, namely, Chinese medicine and diabetic complications, which was also consistent with our research theme. It can be seen that most authors have strong intersection in these two fields. It is worth noting that Professor Liu, Peng’s research is more inclined to diabetic complications (Figure 4B).

**TABLE 2:** Top 10 authors and co-cited authors related to natural products in diabetic kidney disease.

No.	Author	Count	Centrality	Co-cited author	Count	Centrality
1	Li, Ping	22	0.01	Zhang L	79	0.12
2	Liu, Peng	7	0	Navarro-González JF	53	0.11
3	Nan, Zheng	6	0	Alicic RZ	53	0.04
4	Wang, Li	5	0	Forbes JM	40	0.05
5	Deng, Yueyi	4	0	Li Y	37	0.03
6	Feng, Liang	4	0	Zhang Y	35	0.04
7	Han, Lin	4	0	Chen X	34	0.12
8	Liu, Hongfang	4	0	Wang Y	33	0.06
9	Tong, Xiaolin	4	0	Tang GY	32	0.02
10	Xu, Huiqin	4	0	Chen J	32	0.12

Co-cited authors refer to two or more authors who are cited simultaneously in one or more papers. In this study, we obtained 16,679 co-cited authors based on VOSviewer (Figure 4C). Among these authors, Professor Zhang, Lei were the most cited. The followed is Professor Navarro-Gonzalez, JF and Professor Alicic, RZ. The network clearly shows the cooperative relationship of these authors, and three highly-cited authors are relatively independent in three different fields. Among them, Professor Zhang, Lei from the Second Affiliated Hospital of Guangzhou University of Traditional Chinese Medicine is mainly engaged in the research of anti-diabetic nephropathy with traditional Chinese medicine. Professor Navarro-Gonzalez, JF from the Hospital Universitario Nuestra Señora de Candelaria in Spain mainly focuses on the pathological mechanism of chronic kidney disease, while professor Alicic, RZ from the University of Washington School of Medicine prefers clinical research of chronic kidney disease. Further, cluster analysis of these authors’ research directions based on CiteSpace yielded a total of 14 clusters. These studies have both cross fusion and relatively independent directions, indicating that the research content in this field is diversified (Figure 4D).

### 3.4 Journals and co-cited journals analysis

**FIGURE 5:**
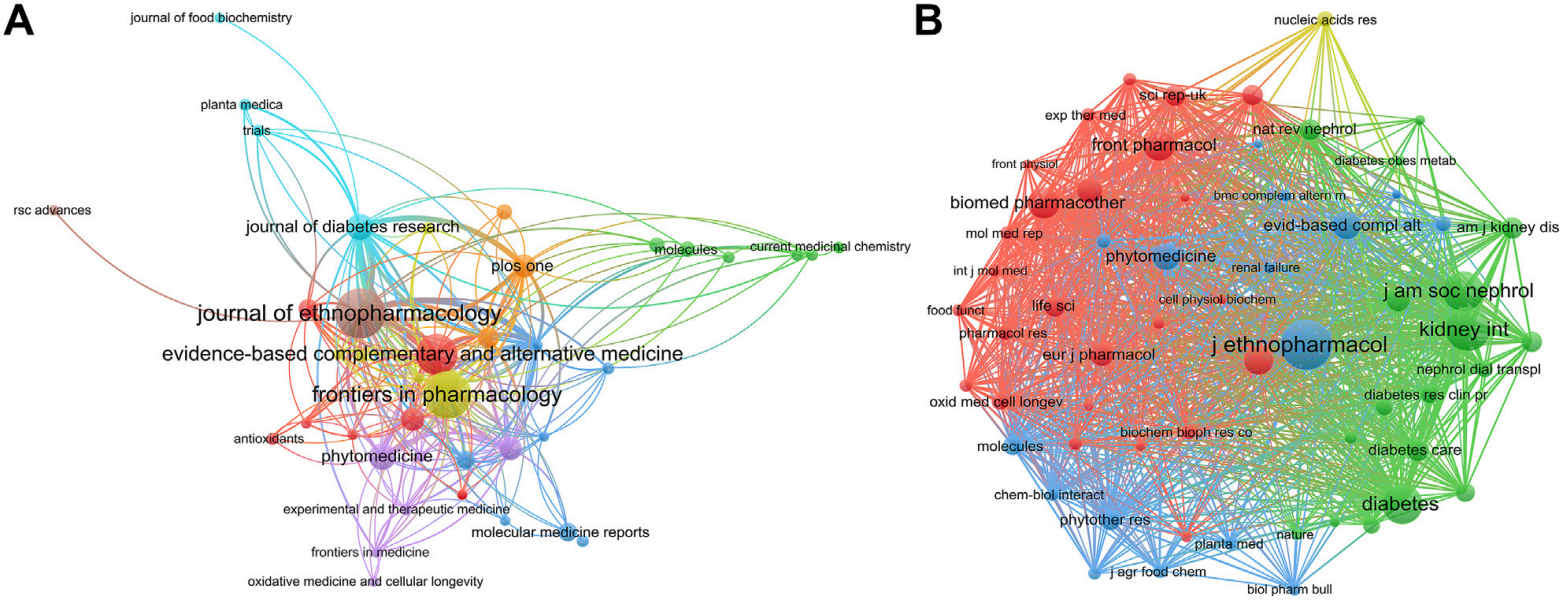
Journals and co-cited journals distribution of publications related to natural products in treating diabetic kidney disease. **(A)** VOSviewer visualization map of different journals. Node sizes are proportional to the number of papers published in these journals, and lines indicate associations between these journals. **(B)** VOSviewer visualization map of different co-cited journals. Node sizes are proportional to the number of papers published in these co-cited journals, and lines indicate associations between them. VOSviewer classifies these co-cited journals into four main categories, denoted by red, blue, green, and yellow.

Based on the analysis of VOSviewer, the 523 papers were published in 178 academic journals (Figure 5; Table 3), of which the Journal of Ethnopharmacology was the most, with 50 papers published and 1,563 citations. Followed by Frontiers in Pharmacology, with 45 papers published and 553 citations. The network shows that these major journals are closely linked. Among the top 10 journals, except the Journal of Diabetes Research and Frontiers in Endocrinology, which mainly publish papers related to clinical diseases, other journals are mainly related to natural products. Besides, in the top 10 journals, there are only two journals’ impact factor greater than 5, namely, Phytomedicine and Biomedicine and Pharmacotherapy, which shows that although there are a large number of researches, the overall quality needs to be improved. However, the impact factor does not fully reflect the quality of a journal. Thus, the average number of citations per literature was further analyzed. Unexpected is, although the total number of papers in Plos One is only 13, the average number of citations per paper is as high as 43.15, followed by Phytomedicine (34.81 citations/paper), Biomedicine and Pharmacotherapy (32.47), Journal of Diabetes Research (31.75) and Journal of Ethnopharmacology (31.26).

**TABLE 3:** Top 10 journals and co-cited journals related to natural products in diabetic kidney disease.

No.	Journal	Documents	Citations	Average citations	If	Co-cited journal	Citations	If
1	Journal of Ethnopharmacology	50	1,563	31.26	4.8	Journal of Ethnopharmacology	785	4.8
2	Frontiers in Pharmacology	45	553	12.29	4.4	Kidney International	672	14.8
3	Evidence-based Complementary and Alternative medicine	35	368	10.51	2.6	Journal of the American Society of Nephrology	596	10.3
4	Journal of Diabetes Research	16	508	31.75	3.6	Diabetes	573	6.2
5	Phytomedicine	16	557	34.81	6.7	Biomedicine and Pharmacotherapy	441	6.9
6	Biomedicine and Pharmacotherapy	15	487	32.47	6.9	Frontiers in Pharmacology	439	4.4
7	Medicine	13	57	4.38	1.3	Plos One	394	2.9
8	Plos One	13	561	43.15	2.9	Evidence-based Complementary and Alternative medicine	380	2.6
9	Frontiers in Endocrinology	12	99	8.25	3.9	Phytomedicine	361	6.7
10	BMC Complementary and Alternative Medicine	10	194	19.40	4.8	International Journal of Molecular Sciences	328	4.9

Among the 4,576 co-cited journals, 24 journals have been cited more than 200 times. Interestingly, the first one is still the Journal of Ethnopharmacology, which has been cited 785 times. This journal, founded in 1979, is a well-established journal related to national pharmacology and plays a decisive role in this field. The second and third ranked journals are Kidney International and the Journal of the American Society of Neurology, with 672 and 596 citations respectively. It is worth noting that these two journals are the top journals in the field of kidney disease. In addition, among the top 10 co-cited journals, there are five journals with impact factor greater than 5, and two journals with impact factor greater than 10. On the one hand, it shows that the papers published in these journals have a strong influence in this field. On the other hand, it indicates that the authors in this field prefer to cite high-quality publications to increase the persuasiveness of the research.

### 3.5 Documents and co-cited references analysis

**FIGURE 6:**
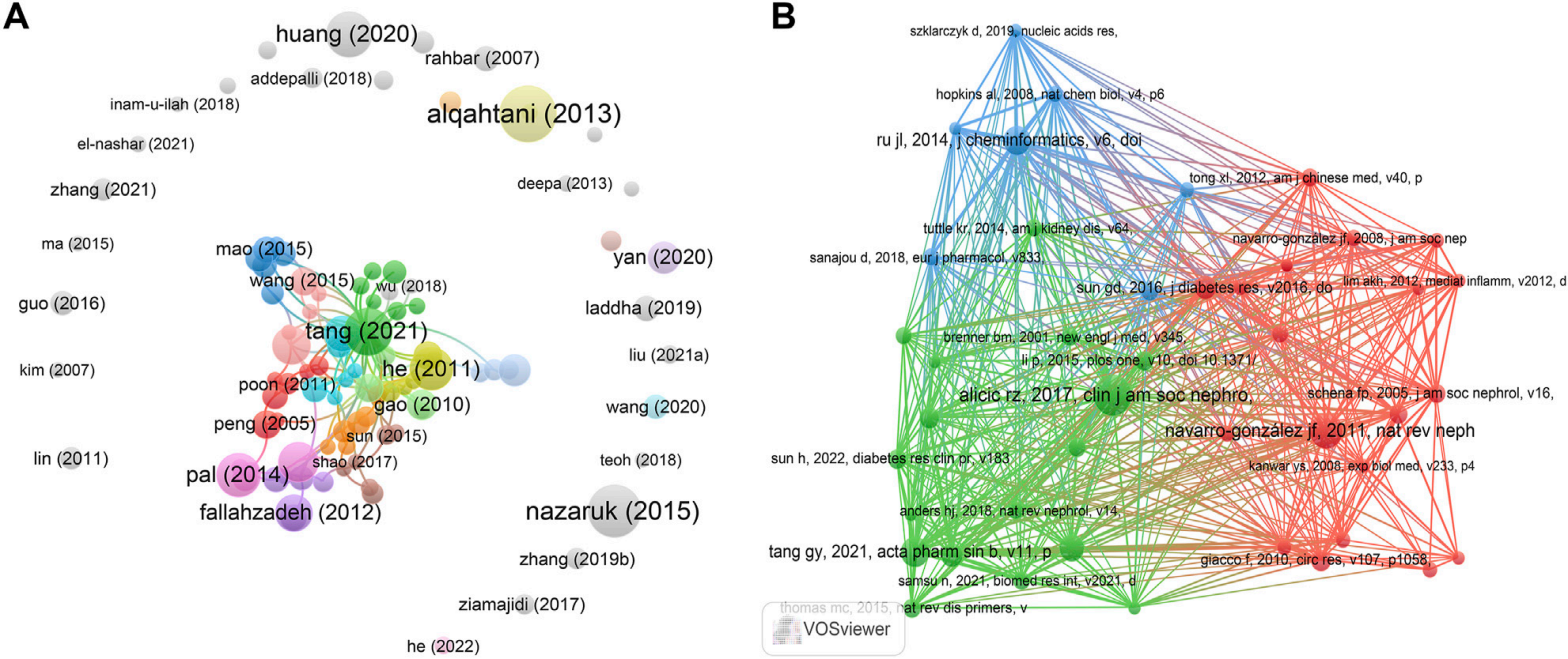
Citations and co-cited references distribution of publications related to natural products in treating diabetic kidney disease. **(A)** VOSviewer visualization map of the publications. Node sizes are proportional to the number of times these publications have been cited, and lines indicate associations between these publications. **(B)** VOSviewer visualization map of co-cited references. Node sizes are proportional to the number of times these co-cited references have been cited, and lines indicate associations between them. VOSviewer classifies these references into three main categories, denoted by red, blue, and green.

We further analyzed the citations of these 523 literature. Table 4 lists the top 10 highly cited papers, all of which have been cited more than 100 times. The paper “The Pentacyclic Triterpenoids in Herbal Medicines and Their Pharmacological Activities in Diabetes and Diabetic Complications” by Professor Alqahtani, A (Alqahtani et al., 2013) from Faculty of Pharmacy of University of Sydney in Australia, ranked first, has been cited 174 times. This review was published in Current Medicinal Chemistry in 2013. The paper “The role of triterpenes in the management of diabetes mellitus and its complications” by Professor Nazaruk, J (Nazaruk and Borzym-Kluczyk, 2015), Medical University of Białystok in Poland, ranked second, and was cited 159 times. This review was published in Phytochemistry Reviews in 2015. In the third place is the paper “Clinical efficacies, underlying mechanisms and molecular targets of Chinese medicines for diabetic nephropathy treatment and management” by Professor Tang, GY (Tang et al., 2021), the University of Hong Kong in China, has been cited 142 times. This review was published in Acta Pharmaceutica Sinica B in 2021. Based on the results of network analysis (Figure 6A), Professor Tang’s highly-cited papers have intersection with other publications, but the papers of Professor Alqahtani, A and Professor Nazaruk, J have basically no intersection with other highly-cited publications. This may be directly related to the author’s country, and China has more research in this field after all.

**TABLE 4:** Top 10 cited documents and co-cited references related to natural products in diabetic kidney disease.

No.	Documents	Citations	Co-cited references	Citations
1	alqahtani (2013)	174	alicic rz, 2017, clin j am soc nephro, v12, p2032, doi 10.2215/cjn.11491116	44
2	nazaruk (2015)	159	navarro-gonzález jf, 2011, nat rev nephrol, v7, p327, doi 10.1038/nrneph.2011.51	38
3	tang (2021)	142	tang gy, 2021, acta pharm sin b, v11, p2749, doi 10.1016/j.apsb.2020.12.020	32
4	huang (2020)	136	ru jl, 2014, j cheminformatics, v6, doi 10.1186/1758-2946-6-13	31
5	pal (2014)	128	umanath k, 2018, am j kidney dis, v71, p884, doi 10.1053/j.ajkd.2017.10.026	28
6	he (2011)	127	selby nm, 2020, diabetes obes metab, v22, p3, doi 10.1111/dom.14007	25
7	xue (2019)	116	sun gd, 2016, j diabetes res, v2016, doi 10.1155/2016/5749857	25
8	li (2011)	108	giacco f, 2010, circ res, v107, p1058, doi 10.1161/circresaha.110.223545	22
9	fallahzadeh (2012)	104	lu zz, 2019, j diabetes res, v2019, doi 10.1155/2019/2697672	22
10	xue (2017)	102	li p, 2015, plos one, v10, doi 10.1371/journal.pone.0126027	21

Similarly, we also analyzed all the 23,829 co-cited references. The most cited paper is “Diabetic Kidney Disease: Challenges, Progress, and Possibilities”, published in Clinical Journal of the American Society of Nephrology by Professor Alicic, RZ in 2017 (Alicic et al., 2017). The review has been cited 44 times in this field. The second is “Inflammatory molecules and pathways in the pathogenesis of diabetic nephropathy” published by Professor Navarro-Gonzalez in Nature Reviews Nephrology in 2011 (Navarro-González et al., 2011), which has been cited 38 times. This result is consistent with that of the co-cited authors. It can be seen that Professor Alicic, RZ and Navarro-Gonzalez have made important contributions in this field. In the third place is the paper “Clinical efficacies, underlying mechanisms and molecular targets of Chinese medicines for diabetic nephropathy treatment and management” by Professor Tang, GY (Tang et al., 2021). This is consistent with the results of highly-cited literature. In the fourth place is Professor Ru, JL of Northwest A&F University, who published “TCMSP: a database of systems pharmacology for drug discovery from herbal medicines” in the Journal of Cheminformatics in 2014 (Ru et al., 2014), and this review has been cited 31 times. According to the network analysis results (Figure 6B), these highly-cited papers are mainly distributed in three different directions. Among them, the papers of professors Alicic, RZ and Tang, GY (green area) are mainly concentrated in the field of clinical research, and the papers of Professor Navarro-Gonzalez (red area) are mainly concentrated in the field of basic research. There are certain intersections between the above two fields. The papers of Professor Ru, JL (blue area) are mainly concentrated in the field of bioinformatics and are relatively independent.

**FIGURE 7:**
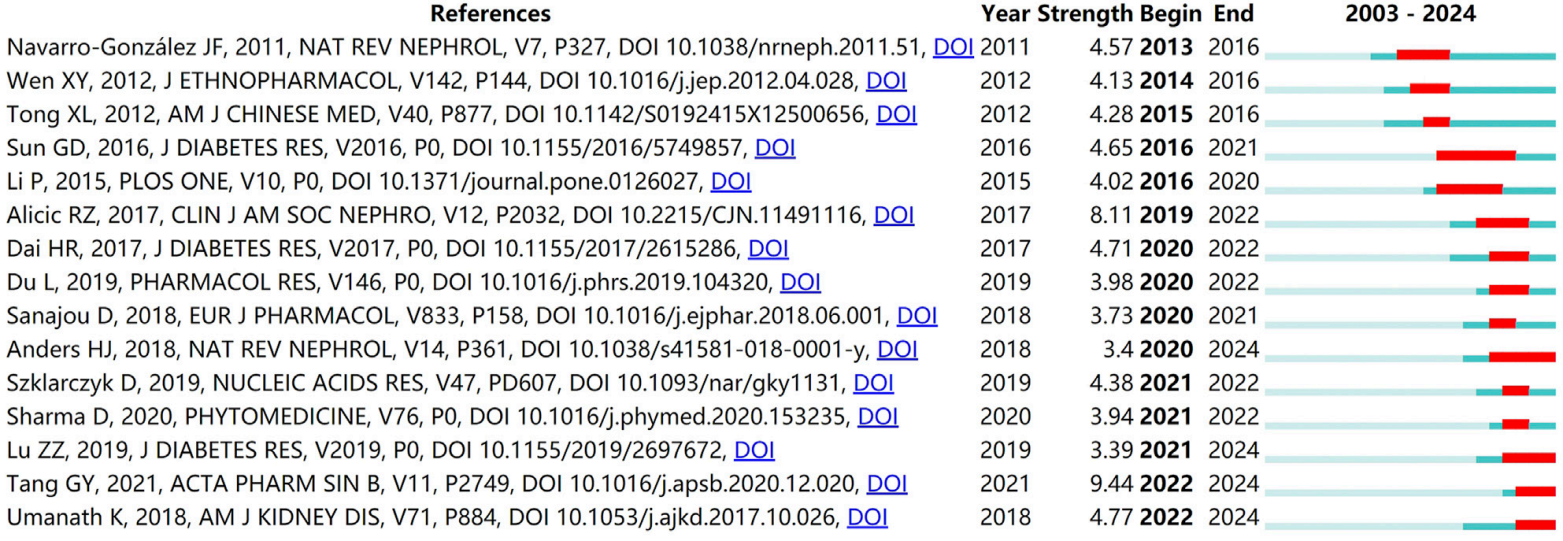
Top 15 co-cited references with the strongest citation bursts related to natural products in treating diabetic kidney disease. The strongest citation burst analysis shows frequently cited literature over a period of time, which are indicated by the red line. The higher the strength of its burst, the higher the heat that was cited during that time period.

In addition, the strongest citation bursts analysis based on CiteSpace reveals the frequently cited literature in a period of time. Figure 7 shows the top 15 strongest citation burst references. As shown in the Figure, the first co-citation outbreak began in 2013 and lasted 4 years. The co-cited paper was published by Professor Navarro-Gonzalez in Nature Reviews Nephrology in 2011, which is consistent with the highly-cited results. The longest citation bursts duration is “Review of Herbal Traditional Chinese Medicine for the Treatment of Diabetic Nephropathy” (Miao et al., 2016), which exhibited sustained citation bursts over a 6-year period. The paper with the strongest bursts is published in Acta Pharmaceutica Sinica B in 2021 by Professor Tang, GY (Tang et al., 2021). The burst strength is as high as 9.44. Combined with the previous results, although the paper was published in 2021, it has been cited 142 times, with an average of 35.5 times a year. It is worth noting that as of 2024, there are still four papers in the state of citation bursts. They are “CKD in diabetes: diabetic kidney disease *versus* nondiabetic kidney disease” (Anders et al., 2018), “The Efficacy and Mechanism of Chinese Herbal Medicine on Diabetic Kidney Disease” (Lu et al., 2019), “Clinical efficacies, underlying mechanisms and molecular targets of Chinese medicines for diabetic nephropathy treatment and management” (Tang et al., 2021), and “Update on Diabetic Nephropathy: Core Curriculum 2018” (Umanath and Lewis, 2018). This means that the related research of herbal medicine against diabetic nephropathy may continue to break out in the future.

### 3.6 Keywords, hotspots and the frontiers analysis

**FIGURE 8:**
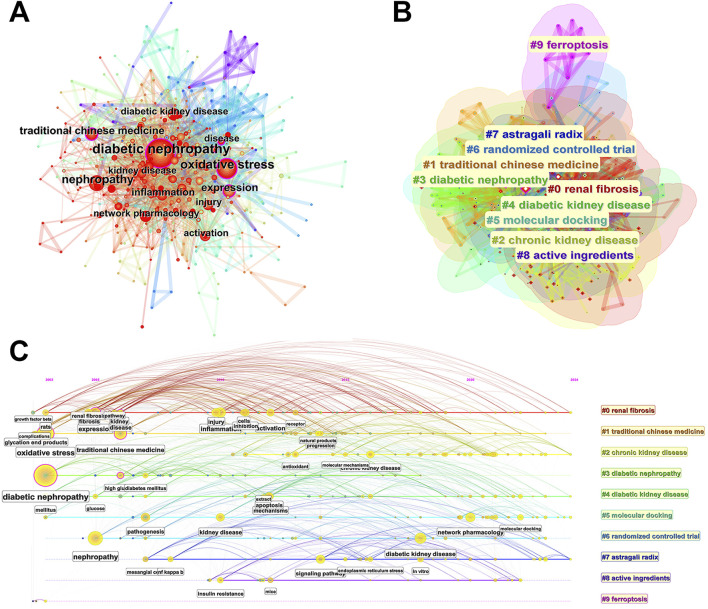
CiteSpace visualization map of keywords related to natural products in treating diabetic kidney disease. **(A)** CiteSpace visualization map of different keywords. The nodes represent the keywords, and the larger the diameter, the higher the frequency of the keyword. Lines represent connections between keywords. **(B)** Cluster analysis of the keywords based on their category. Cluster analysis is used to analyze keywords according to the subject classification in order to have a clearer understanding of the research field. In this study, all keywords were divided into 10 main clusters, represented by different colors and umbrella shapes. **(C)** Timeline chart of different keywords. The timeline chart is to arrange all keywords according to time and category. The horizontal coordinate represents the time axis, and the vertical coordinate represents the 10 clusters. Based on the timeline chart, the evolution law of keywords in each category over time can be more clearly observed, so as to judge the trend change of research hotspots.

Based on the analysis of keywords, we can pry the hot research direction from big data, so as to systematically understand the specific research field. Figure 8A analyzes the relationship of keywords in these 523 publications based on CiteSpace, and Table 5 lists the top 20 high-frequency keywords. The results showed that “diabetic nephropathy” occurred 255 times with a high centrality (0.12), followed by “oxidative stress” and “nephropathy”, with a frequency of 155 and 118 times, respectively. In addition, “expression” and “traditional Chinese medicine” also with high centrality (0.14 and 0.12), and the occurrence times were 72. From the perspective of network analysis, these keywords have strong intersection, indicating that the research direction in this field is wide.

**TABLE 5:** Top 20 keywords related to natural products in diabetic kidney disease.

No.	Keywords	Count	Centrality	No.	Keywords	Count	Centrality
1	diabetic nephropathy	255	0.12	11	network pharmacology	43	0.02
2	oxidative stress	155	0.14	12	disease	43	0.13
3	nephropathy	118	0.09	13	kidney	39	0.08
4	expression	72	0.14	14	mechanisms	38	0.05
5	traditional Chinese medicine	72	0.12	15	rats	38	0.08
6	inflammation	67	0.04	16	fibrosis	37	0.04
7	diabetic kidney disease	52	0.02	17	glycation end products	37	0.09
8	activation	52	0.07	18	pathogenesis	35	0.04
9	kidney disease	44	0.07	19	signaling pathway	35	0.04
10	injury	44	0.03	20	apoptosis	34	0.07

On the basis of network analysis, we have carried out cluster analysis on these high-frequency keywords according to subject classification, and obtained a total of 10 main directions (Figure 8B), namely, “renal fibrosis”, “traditional Chinese medicine”, “chronic kidney disease”, “diabetic nephropathy”, “diabetic kidney disease”, “molecular docking”, “randomized controlled trial”, “astragali radix”, “active ingredients”, and “ferroptosis”. Except for ferroptosis, there are strong intersections in most other clusters. Renal fibrosis is a common pathological feature of various chronic kidney diseases, including diabetic kidney, which eventually progresses to end-stage renal disease. Renal fibrosis is clustered as a core research direction, indicating that in the research of natural products against DKD, many studies are involved in the exploration of the pathological mechanism of fibrosis. In addition, it is noteworthy that Astragali Radix is also clustered as a core research direction. Astragali Radix has been used in China and East Asian countries for thousands of years. It is also widely used in diabetes and chronic kidney disease. Modern pharmacological studies have shown that the main components of Astragali Radix include Astragalus polysaccharides, Astragaloside IV, etc. The results of this study suggest that Astragali Radix is more concerned by researchers in the research of natural products against DKD. It also shows that Astragali Radix may has great potential and clinical value in the treatment of DKD.

**FIGURE 9:**
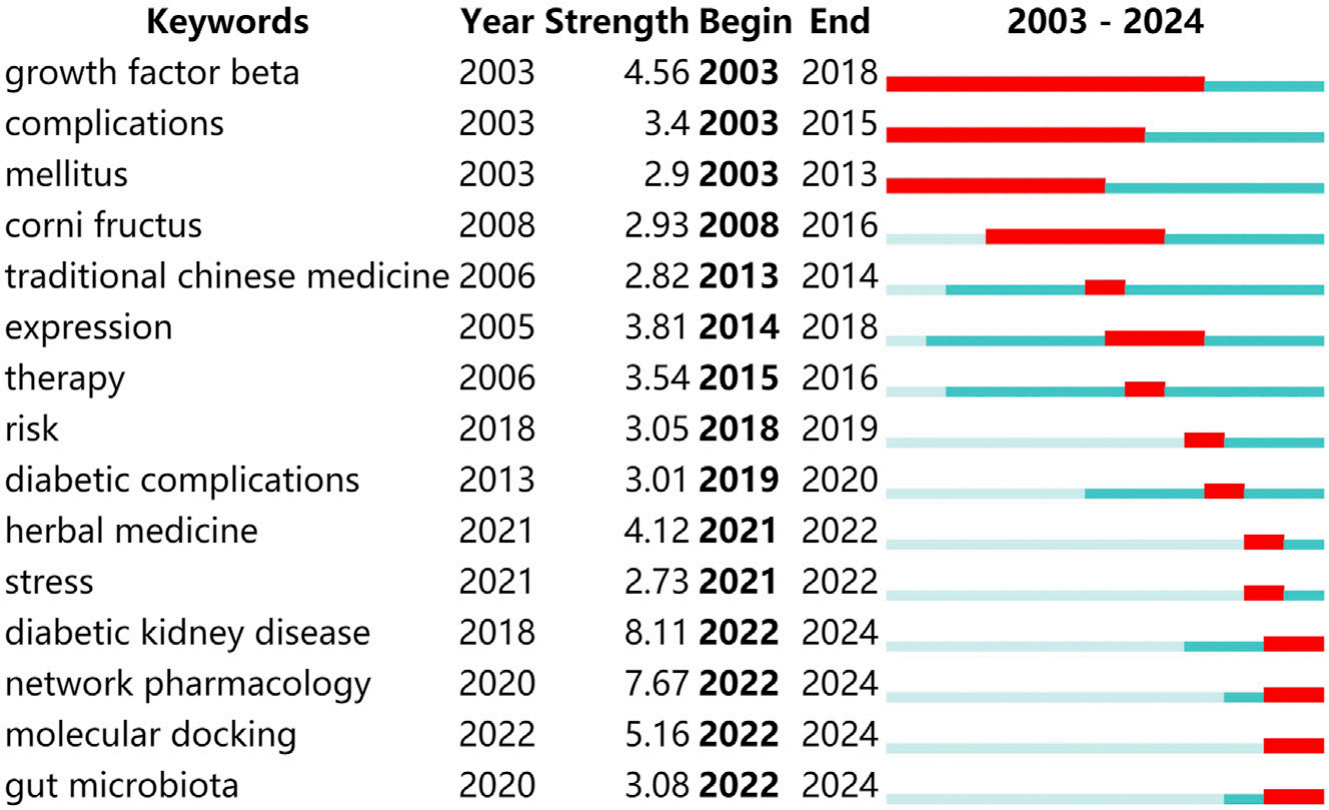
Top 15 keywords with the strongest citation bursts related to natural products in treating diabetic kidney disease. The strongest citation burst analysis shows frequently keywords over a period of time, which are indicated by the red line. The higher the strength of its burst, the more frequently the keyword is noticed during that time period. This graph visually shows the changing trends of research hotspots in the field over time.

The timeline chart can connect one or more events in chronological order to form an intuitive description. Based on the interaction between keywords in a specific field, timeline chart aims to help explore the evolutionary trajectory and stage characteristics of a field. Figure 8C is the keyword timeline drawn by CiteSpace, which directly reflects the research hotspots and development paths of natural products in treating DKD from the time dimension. It can be seen from the figure that the distribution of these keywords can be divided into two time periods. From 2003 to 2015, the research mainly focused on the conventional pathological and pharmacological mechanism, and the keywords involved include “growth factor beta”, “inflammation”, “oxidative stress”, “apoptosis”, etc. From 2015 to 2024, the research is more inclined to the field of bioinformatics, and the keywords involved include “network pharmacology”, “molecular docking”, etc. This is closely related to the rapid development of big data in recent years. Due to the multi-component and multi-target characteristics of traditional Chinese medicine, the conventional research is difficult to fully reveal the complex mechanism of traditional Chinese medicine. Based on the big data system, network pharmacology could associate the multi-component of traditional Chinese medicine with the multi-target of disease from the overall level, which coincides with the theory of traditional Chinese medicine (Li, 2022). This result is consistent with the strongest citation bursts analysis of keywords (Figure 9), and in which “network pharmacology”, “molecular docking” and “gut microbiota” showed an explosive trend from 2022 to 2024. It is expected that this research field will continue to become a hot spot in the next few years.

## 4 Research gaps and prospects

### 4.1 Broader and closer cooperation is expected

From the perspective of this paper, the research in this field is relatively concentrated and the communication is not extensive enough at the level of countries, institutions and authors. At the national level, most of the studies are concentrated in China, with a small number of studies in East Asian countries such as India, South Korea, and Japan. The main reason for this phenomenon is related to the widespread use of herbal medicine, especially Chinese medicine, in East Asian countries. Although there is cooperation among these countries, the vast majority of cooperation is mainly within the country, and the cooperation between countries is less. At the institutional level, the top research institutions are all in China. And the cooperation among these institutions shows regional differences. From the author level, the top ten authors are all Chinese scholars, which is consistent with the previous conclusion. It is worth noting that these authors are mainly distributed in the two fields of natural product research and DKD research. In the context of the rapid development of interdisciplinary disciplines, the communication between the two fields needs to be strengthened. Currently, the pace of new drug development has significantly slowed down, while herbal medicine has demonstrated unique advantages in the treatment of diseases, especially chronic ones (Li and Vederas, 2009). An increasing number of scholars have recognized that natural products constitute a vast treasure trove for new drug discovery (Chopra and Dhingra, 2021). Therefore, strengthening exchanges and cooperations among authors, institutions, and countries, and engaging more scholars, especially those from non-East Asian regions, to focus on and study herbal medicine, will provide new strategies and ideas for the future clinical treatment and drug development of DKD.

### 4.2 The quality of research still needs to be improved

Based on the results in this article, there have been 523 papers published in this field over the past 25 years, with an average of more than 20 papers per year. Notably during the recent quadrennium (2021–2024), the publications have exceeded 60 articles every year, which reflects the increasing popularity of research in this field year by year. However, the results also show that the quality of research in this field needs to be improved, which is reflected in two aspects. For one thing, most of the studies are published in journals related to complementary and alternative medicine or comprehensive journals, with a generally low impact factor and a lack of papers in authoritative top-tier journals. On the contrary, the journals co-cited by these papers have higher impact factors. For another, among the 523 papers, only 10 papers achieved citation counts exceeding 100, accounting for only 1.9%; and only 43 papers have been cited 50–100 times, accounting for only 8.2%. However, 470 papers have been cited less than 50 times, accounting for as high as 89.9%. There may be two reasons for the above results. First, due to the complex chemical composition of herbal medicine, it is difficult to gain full recognition internationally (Lin et al., 2018). On the other hand, the depth of research in this field is not enough. Most of these studies tend to focus on the pharmacodynamic effects of a natural product, although they involve the mechanism of treating DKD, but lack in-depth exploration of the molecular biological mechanism. In addition, most of the current research is conducted at the animal and cellular levels, lacking clinical verification, and these studies still have a long way to go before the successful development of drugs. Therefore, it is of great significance to strengthen the exploration of the mechanism emphasis on clinical research, in order to improve the quality of research and promote the development of new drugs for anti-DKD.

### 4.3 Computer-based data analysis has become a trend and hot spot

Our findings reveal that the keywords in this field have demonstrated a staged explosive trend over time. Particularly notable is that systems biology and bioinformatics research represented by network pharmacology have emerged as new hotspots during the recent triennial period (2022–2024).

The ideal situation in modern medicine is to apply a drug to a specific target, corresponding to a specific gene or a particular disease, this is difficult to achieve in real life. With the continuous deepening of systems biology and pharmacological research, the modern medical technology system and research methods have been constantly refined. British pharmacologist Hopkins AL first proposed the concept of “network pharmacology” in 2007(Hopkins, 2007), defining it as a pharmacological sub-discipline that utilizes network methods to analyze the “multi-component composition, multi-target interaction, multi-pathway modulation” synergistic interaction relationships between drugs, diseases, and targets. To date, network pharmacology has gradually evolved into a mature interdisciplinary field. On the basis of the data of systems biology, network pharmacology integrates the three aspects of targets, drugs, and diseases (Wang et al., 2024). Through the analysis of biological complex networks, valuable information is obtained and verified by experimental results. Its scientific and reasonable application to the research of natural products is conducive to promoting the development of herbal medicine in clinical settings.

However, the following points should be taken into consideration in future research: First, enhancing the standardization of databases. The research of network pharmacology is based on the acquisition of big data. However, the data quality and scale in current databases vary and are constantly being improved and updated (Zhang et al., 2019). A unified standard should be used to make the research of network pharmacology more scientific and standardized (Li, 2021). Second, strengthening the verification of prediction results. The acquisition of targets for natural product components is typically based on databases and computer predictions. These predicted components and targets often lack the forms of action in biological systems, and their authenticity and accuracy have not been verified (Nogales et al., 2022). In related research, network pharmacology should serve as a preliminary screening tool, and the reliable verification of obtained targets is crucial for improving the quality of research. Third, intensifying the integration with artificial intelligence. With the increasing maturity of artificial intelligence algorithms and models such as graph neural networks and convolutional neural networks, research in the field of molecular mechanisms of biological diseases has deepened, and related applications have gradually carried out and achieved relevant results (Abdul Raheem and Dhannoon, 2024; Edeh et al., 2022). The application of artificial intelligence in the field of natural products can summarize the correlation rules contained in massive data with high precision and efficiency, which is highly beneficial for accelerating the speed of new drug research and development (Zhang et al., 2024).

## 5 Limitations

Although this paper systematically analyzed the research status of natural products in diabetic nephropathy, there are still some limitations in the current research. First, since the bibliometrics analysis software lacked accuracy in identifying and analyzing non-English literature, and mixed analysis would interfere with the results. The language of the literature included in this study is limited to English, and five non-English reports were excluded, which may produce potential bias. In addition, China is a key area for natural medicine research, so there are a large number of reports in this field in Chinese literature databases (such as CNKI). However, given that this is an international paper, the study was designed to include only English-language literature to ensure the quality of the included literature and to increase readability for researchers worldwide. Second, in this study, Excel, CiteSpace and VOSviever were used to analyze and visualize the information in the literature. Although these tools are mainstream in bibliometrics, more robust tools such as R, Python, and artificial intelligence are now mature, and will be more intelligent and efficient in data extraction, sorting and analysis. It is regrettable that due to the limitations of current software’s capabilities, these tools were not used for the time being. We believe that the widespread application of these tools in big data analytics will be an inevitable trend. Third, we mainly conducted statistics on countries, institutions and authors based on the number of published papers in this field. However, the number of publications alone does not fully reflect the research intensity of these countries/institutions/authors, and many factors could affect the number of publications. In this study, due to the limited information available, there was no standardized processing of these data. More systematic data collection, standardized data processing and intelligent data analysis will be the future development direction of bibliometrics.

## 6 Conclusion

In recent years, the application of natural products in chronic diseases has gradually received more attention, and more scholars have paid attention to and invested in research on herbal medicine for DKD. Therefore, there is an urgent need to systematically analyze and summarize the latest research progress in this field. This study obtained 523 relevant literature based on the WOS database and conducted visualization analysis of these papers using bibliometric methods. The research findings are as follows: 1) The number of publications in this field has increased year by year, reflecting the research heat; 2) From the analysis of countries, institutions, and authors, China has an absolute advantage in this field, and Professor Li Ping has published the most papers. 3) The Journal of Ethnopharmacology is the most popular journal, and Alqahtani (2013) is the most cited literature. 4) Bioinformatics research, represented by network pharmacology, has become a new research hotspot in this field. It should be noted that our purpose is not only to comprehensively introduce the research progress of natural products for DKD, but also to reveal the blank spots and shortcomings of previous research and provide insights into the possible research focus of the future.

## Data Availability

The original contributions presented in the study are included in the article/supplementary material, further inquiries can be directed to the corresponding authors.

## References

[B1] Abdul Raheem A. K. Dhannoon B. N. (2024). A novel deep learning model for drug-drug interactions. Curr. Comput.-Aid. Drug. 20 (5), 666–672. 10.2174/0115734099265663230926064638 38804324

[B2] Alicic R. Z. Rooney M. T. Tuttle K. R. (2017). Diabetic kidney disease: challenges, progress, and possibilities. Clin. J. Am. Soc. Nephro. 12 (12), 2032–2045. 10.2215/CJN.11491116 PMC5718284 28522654

[B3] Alqahtani A. Hamid K. Kam A. Wong K. H. Abdelhak Z. Razmovski-Naumovski V. (2013). The pentacyclic triterpenoids in herbal medicines and their pharmacological activities in diabetes and diabetic complications. Curr. Med. Chem. 20 (7), 908–931. 10.2174/092986713805219082 23210780

[B4] Anders H. J. Huber T. B. Isermann B. Schiffer M. (2018). CKD in diabetes: diabetic kidney disease versus nondiabetic kidney disease. Nat. Rev. Nephrol. 14 (6), 361–377. 10.1038/s41581-018-0001-y 29654297

[B5] Chen C. (2006). CiteSpace II: detecting and visualizing emerging trends and transient patterns in scientific literature. J. Am. Soc. Inf. Sci. Technol. 57 (3), 359–377. 10.1002/asi.20317

[B6] Chopra B. Dhingra A. K. (2021). Natural products: a lead for drug discovery and development. Phytother. Res. 35 (9), 4660–4702. 10.1002/ptr.7099 33847440

[B7] DeFronzo R. A. Reeves W. B. Awad A. S. (2021). Pathophysiology of diabetic kidney disease: impact of SGLT2 inhibitors. Nat. Rev. Nephrol. 17 (5), 319–334. 10.1038/s41581-021-00393-8 33547417

[B8] Edeh M. O. Dalal S. Dhaou I. B. Agubosim C. C. Umoke C. C. Richard-Nnabu N. E. (2022). Artificial Intelligence-Based ensemble learning model for prediction of hepatitis c disease. Front. Public Health 10, 892371. 10.3389/fpubh.2022.892371 35570979 PMC9092454

[B9] Georgianos P. I. Vaios V. Eleftheriadis T. Papachristou E. Liakopoulos V. (2023). Therapeutic advances in diabetic kidney disease. Int. J. Mol. Sci. 24 (3), 2803. 10.3390/ijms24032803 36769113 PMC9917247

[B10] Gnudi L. Coward R. J. M. Long D. A. (2016). Diabetic nephropathy: perspective on novel molecular mechanisms. Trends Endocrinol. and Metabolism 27 (11), 820–830. 10.1016/j.tem.2016.07.002 27470431

[B11] Herrington W. G. Haynes R. (2024). Diabetic kidney disease-semaglutide flows into the mainstream. N. Engl. J. Med. 391 (2), 178–179. 10.1056/NEJMe2406408 38986062

[B12] Hicks D. Wouters P. Waltman L. de Rijcke S. Rafols I. (2015). Bibliometrics: the leiden manifesto for research metrics. Nature 520 (7548), 429–431. 10.1038/520429a 25903611

[B13] Hopkins A. L. (2007). Network pharmacology. Nat. Biotechnol. 25 (10), 1110–1111. 10.1038/nbt1007-1110 17921993

[B14] Hu Q. Jiang L. Yan Q. Zeng J. Ma X. Zhao Y. (2023). A natural products solution to diabetic nephropathy therapy. Pharmacol. Ther. 241, 108314. 10.1016/j.pharmthera.2022.108314 36427568

[B15] Kanasaki K. Ueki K. Nangaku M. (2024). Diabetic kidney disease: the kidney disease relevant to individuals with diabetes. Clin. Exp. Nephrol. Online ahead print 28, 1213–1220. 10.1007/s10157-024-02537-z PMC11621156 39031296

[B16] Li J. W. Vederas J. C. (2009). Drug discovery and natural products: end of an era or an endless frontier. Science 325 (5937), 161–165. 10.1126/science.1168243 19589993

[B17] Li S. (2021). Network pharmacology evaluation method Guidance-Draft. World J. Tradit. Chin. Med. 7 (1), 148. 10.4103/wjtcm.wjtcm_11_21

[B18] Li S. (2022). Network pharmacology. Berlin: Tsinghua University Press.

[B19] Liao H. Wang X. Wang X. Zhang M. Zhang Y. Huang S. (2024). Organohalide respiration: retrospective and perspective through bibliometrics. Front. Microbiol. 15, 1490849. 10.3389/fmicb.2024.1490849 39777152 PMC11703978

[B20] Lin A. X. Chan G. Hu Y. Ouyang D. Ung C. O. L. Shi L. (2018). Internationalization of traditional Chinese medicine: current international market, internationalization challenges and prospective suggestions. Chin. Med. 13 (9), 9. 10.1186/s13020-018-0167-z 29449877 PMC5807832

[B21] Lu Z. Zhong Y. Liu W. Xiang L. Deng Y. (2019). The efficacy and mechanism of Chinese herbal medicine on diabetic kidney disease. J. Diabetes Res. 2019, 2697672. 10.1155/2019/2697672 31534972 PMC6732610

[B22] Miao L. Dong W. Zou H. Dong C. Guo Q. Cui W. (2016). Review of herbal traditional Chinese medicine for the treatment of diabetic nephropathy. J. Diabetes Res. 2016, 1–18. 10.1155/2016/5749857 PMC4662991 26649322

[B23] Navarro-González J. F. Mora-Fernández C. Muros De Fuentes M. García-Pérez J. (2011). Inflammatory molecules and pathways in the pathogenesis of diabetic nephropathy. Nat. Rev. Nephrol. 7 (6), 327–340. 10.1038/nrneph.2011.51 21537349

[B24] Nazaruk J. Borzym-Kluczyk M. (2015). The role of triterpenes in the management of diabetes mellitus and its complications. Phytochem. Rev. 14 (4), 675–690. 10.1007/s11101-014-9369-x 26213526 PMC4513225

[B25] Nogales C. Mamdouh Z. M. List M. Kiel C. Casas A. I. Schmidt H. H. H. W. (2022). Network pharmacology: curing causal mechanisms instead of treating symptoms. Trends Pharmacol. Sci. 43 (2), 136–150. 10.1016/j.tips.2021.11.004 34895945

[B26] Ru J. Li P. Wang J. Zhou W. Li B. Huang C. (2014). TCMSP: a database of systems pharmacology for drug discovery from herbal medicines. J. Cheminform 6, 13. 10.1186/1758-2946-6-13 24735618 PMC4001360

[B27] Tang G. Li S. Zhang C. Chen H. Wang N. Feng Y. (2021). Clinical efficacies, underlying mechanisms and molecular targets of Chinese medicines for diabetic nephropathy treatment and management. Acta Pharm. Sin. B 11 (9), 2749–2767. 10.1016/j.apsb.2020.12.020 34589395 PMC8463270

[B28] Tian S. Chen M. (2024). The mechanisms and drug therapies of colorectal cancer and epigenetics: bibliometrics and visualized analysis. Front. Pharmacol. 15, 1466156. 10.3389/fphar.2024.1466156 39268463 PMC11391208

[B29] Umanath K. Lewis J. B. (2018). Update on diabetic nephropathy: core Curriculum 2018. Am. J. Kidney Dis. 71 (6), 884–895. 10.1053/j.ajkd.2017.10.026 29398179

[B30] Van Eck N. J. Waltman L. (2010). Software survey: VOSviewer, a computer program for bibliometric mapping. Scientometrics. 84 (2), 523–538. 10.1007/s11192-009-0146-3 20585380 PMC2883932

[B31] Wang X. Wang Y. Yuan T. Wang H. Zeng Z. Tian L. (2024). Network pharmacology provides new insights into the mechanism of traditional Chinese medicine and natural products used to treat pulmonary hypertension. Phytomedicine 135, 156062. 10.1016/j.phymed.2024.156062 39305743

[B32] Wu D. Karimi-Maleh H. Liu X. Fu L. (2023). Bibliometrics analysis of research progress of electrochemical detection of tetracycline antibiotics. J. Anal. Methods Chem. 2023, 6443610–6443614. 10.1155/2023/6443610 36852208 PMC9966827

[B33] Yarmohammadi F. Wallace Hayes A. Karimi G. (2024). Molecular mechanisms involved in doxorubicin-induced cardiotoxicity: a bibliometrics analysis by VOSviewer. Naunyn-Schmiedeberg's Archives Pharmacol. 397 (4), 1971–1984. 10.1007/s00210-023-02773-2 37812241

[B34] Zhang P. Zhang D. Zhou W. Wang L. Wang B. Zhang T. (2024). Network pharmacology: towards the artificial intelligence-based precision traditional Chinese medicine. Brief. Bioinform. 25 (1), bbad518–12. 10.1093/bib/bbad518 PMC10777171 38197310

[B35] Zhang R. Zhu X. Bai H. Ning K. (2019). Network pharmacology databases for traditional Chinese medicine: review and assessment. Front. Pharmacol. 10, 123. 10.3389/fphar.2019.00123 30846939 PMC6393382

[B36] Zheng S. Xu Y. Zhang Y. Long C. Chen G. Jin Z. (2024). Efficacy and safety of traditional Chinese medicine decoction as an adjuvant treatment for diabetic nephropathy: a systematic review and meta-analysis of randomized controlled trials. Front. Pharmacol. 15, 1327030. 10.3389/fphar.2024.1327030 38783937 PMC11111926

[B37] Zheng W. Guo J. Liu Z. (2021). Effects of metabolic memory on inflammation and fibrosis associated with diabetic kidney disease: an epigenetic perspective. Clin. Epigenetics. 13 (1), 87. 10.1186/s13148-021-01079-5 33883002 PMC8061201

[B38] Zhong S. Wang N. Zhang C. (2024). Podocyte death in diabetic kidney disease: potential molecular mechanisms and therapeutic targets. Int. J. Mol. Sci. 25 (16), 9035. 10.3390/ijms25169035 39201721 PMC11354906

[B39] Zhou T. Fang Y. Tian T. Wang G. (2024). Pathological mechanism of immune disorders in diabetic kidney disease and intervention strategies. World J. Diabetes 15 (6), 1111–1121. 10.4239/wjd.v15.i6.1111 38983817 PMC11229953

